# The Rise of Blood Pressure in Later Life

**Published:** 1903-01-31

**Authors:** 


### THE RISE OF BLOOD PRESSURE IN LATER LIFE.

At the last meeting of the Royal Medical and Chirurgical Society Professor Clifford Allbutt read a paper on the rise of blood pressure in later life. The increased pressure in Bright's disease was, he said, perhaps the best known instance of such a rise, but the change in question was by no means confined to that disease. It was generally assumed that arterial disease was necessarily attended with a rise of blood pressure, many kinds of arterial disease being comprehended or confused under the title arterio-sclerosis. Such, however, was far from being the case: it was not true for example of many of the acuter affections of the arteries, while in many of the chronic, and even of the involutionary forms, however extreme, a high blood pressure was conspicuously absent. The more senile forms of arterial disease might then be divided into those in which blood pressure was raised and those in which it was normal or even low. The first kind—those with high pressure—tended to death by apoplexy; the latter tended, often after length of years, rather to obliterative changes in the cerebral arteries with softening. As to the cause of the raised blood pressure this must depend upon two antecedents, singly or in combination—namely, diminution of the calibre and therefore of the capacity of the arterial system, and increased viscosity of the blood. Now it was not proved, or even probable, that the loss of elasticity in the vessels met with in later life, always or even generally led to loss of capacity, and Professor Allbutt suggested that the tendency to rise of pressure often seen in elderly people, especially in such as had led a sedentary life, with positive or relative excess of food, depended rather on increase of viscosity of the blood. If this were so, and if as he suggested arterio-sclerosis was a result rather than a cause of high pressure the tendency to such rises of pressure, and the causes of such increased viscosity of the blood as might produce it should be carefully watched. As to the nature of blood changes, by which this rise of pressure was produced, it was not possible to say much beyond the negative statement that there was no definite reason to allege that it was of the nature of gout, whatever gout might be, although it might well depend on some perversion of assimilation or excretion having an affinity to that condition. The important matter, however, was to detect in time the morbid tendency to rise of blood pressure. The finger would tell much, but such estimates of pressure very properly had not commanded ready acceptance. The personal equation of the observer entered too far into such observations; but by the useful instruments invented by von Basch, Hill and Barnard, and Oliver, much useful information could be obtained as to the blood-pressure, and if by these means a morbid disposition to rise of pressure were detected in time the condition was remediable. The means to be adopted in the treatment of such a case were chiefly deobstruant, dietetic, and gymnastic; the necessary change in the habits of life being of course permanently imposed upon the patient. If, however, the conditions were not detected till the strain had resulted in a new "set" of the vascular tissues, then, even if palliation were possible, cure was out of the question.

